# Can we taste extensiveness? Linking production concepts of extensification factors to the eating quality and consumer liking of chicken breast meat

**DOI:** 10.1016/j.psj.2026.106379

**Published:** 2026-01-03

**Authors:** Seren Yigitturk, Marlene Schou Grønbeck, Shai Barbut, Line Ahm Mielby, Birthe Steenberg, Sara Wilhelmina Erasmus

**Affiliations:** a Food Quality and Design, Wageningen University & Research, P.O. Box 17, 6700 AA, Wageningen, the Netherlands; b Danish Technological Institute, Food and Production, Gregersensvej 1, 2630 Taastrup, Denmark; c Adaptation Physiology Group, Department of Animal Sciences, Wageningen University & Research, Wageningen, the Netherlands; d Department of Food Science, University of Guelph, Guelph N1G 2W1 Ontario, Canada; e AVEC Association of Poultry Processors and Poultry Trade in the EU Countries, Rue du Luxembourg 47-51, B-1050 Brussels, Belgium

**Keywords:** Broiler meat quality, Consumer acceptance, Juiciness, Physicochemical quality, Sensory analysis

## Abstract

Broiler production in Europe is exploring extensification factors such as slower-growing genotypes, dietary fibre supplementation, increased space allowance and environmental enrichment. While these strategies aim to balance productivity with environmental sustainability and animal welfare, how their combined system-level profiles influence eating quality and consumer liking remains unclear. This study evaluated chicken breast fillets from eight production concepts, each representing a distinct system-level combination of genetics, diet, space allowance and enrichment across higher-welfare non-organic and organic systems. Production concepts were implemented in the Netherlands and Germany as part of a European research consortium specializing in higher-welfare and organic broiler production systems. Breast fillets were characterized using physicochemical quality measurements and trained descriptive sensory profiling. In addition, a subset of concepts with distinct sensory profiles was evaluated in a consumer test. The descriptive sensory profiling as well as the consumer test was conducted in Denmark by the designated consortium partner due to their specific competencies. Across concepts, when moisture content was similar, lean, protein-dense breast meat with higher firmness was perceived as less juicy when roasted, despite exhibiting high water-holding capacity. This suggests a dual contribution of fat content and muscle structural properties to oral juiciness. Concepts that combined lower first-bite hardness with higher sensory tenderness and juiciness also achieved higher consumer liking of juiciness, underscoring the central role of the tenderness-juiciness axis in consumer acceptance. Colour differences in breast fillets were detected instrumentally and by trained panellists, but these contrasts were not reflected in consumer appearance liking, indicating that visual cues in cooked meat were less influential than juiciness. Overall, genotype emerged as the principal driver of eating quality. System-level profiles of extensification factors shaped product characteristics, but consumer liking differences were modest and mainly linked to juiciness.

## Introduction

Chicken meat is an important animal protein source in Europe, with annual production reaching about 14 million tons in 2024 (AVEC, 2025). Although poultry has a substantially lower environmental footprint than other livestock (OECD/FAO, 2025), the poultry industry faces increasing pressure to improve sustainability and animal welfare while maintaining eating quality (de Araújo et al., 2022).

Intensive farming systems are generally thought to have higher productivity (Bilotto et al., 2024). However, their implementation has in some cases had deleterious environmental effects and has been identified as a major driver of biodiversity loss through land-use change, feed production and associated ecosystem pressures (Bilotto et al., 2024; Pereira et al., 2025). In response to these challenges, the Food and Agriculture Organization (FAO) has called for a transition toward livestock farming systems that balance productivity with environmental sustainability and animal welfare (Doyle et al., 2025). This call is embedded within a One Health perspective, which frames the welfare of animals used in food production not only as an ethical responsibility but also as integral to resilient food systems, human health and sustainable livelihoods (Doyle et al., 2025). At the same time, the continuing rise in global demand for animal protein (OECD/FAO, 2025) has further stimulated interest in extensification factors for broiler farming (Marchewka et al., 2023). These factors aim to improve animal welfare beyond physical health alone, encompassing animals’ nutritional, husbandry and behavioural needs (Doyle et al., 2025). Approaches include the use of slower-growing genotypes, provision of environmental enrichment, increased space allowance, dietary supplementation and, particularly in organic systems, outdoor access (Ludwiczak et al., 2023; Marchewka et al., 2023).

Among these strategies, the use of alternative genotypes for meat such as dual-purpose breeds and male-layer strains has gained attention as a means to avoid the culling of day-old male chicks and thereby address an important ethical concern (de Haas et al., 2021). These genotypes grow more slowly, reach older slaughter ages and show reduced production efficiency, which raises questions regarding their economic viability and long-term sustainability (Mueller et al., 2018; Siekmann et al., 2018; de Haas et al., 2021). At the product level, their distinct characteristics may influence sensory quality and consumer acceptance (Siekmann et al., 2018; de Haas et al., 2021).

Eating quality is multidimensional and arises from interactions between product physicochemical characteristics and human sensory processing (Troy and Kerry, 2010). Further, consumer liking is shaped by complex processes beyond immediate sensory input, including familiarization, expectations, prior experience and the context of consumption, among others (Deliza and MacFie, 1996; Borgogno et al., 2015). Physicochemical measurements provide objective descriptors of meat characteristics (Mir et al., 2017; Barbut and Leishman, 2022). Sensory and consumer evaluations determine how these characteristics are perceived and valued (Murray et al., 2001; de Araújo et al., 2022). Integrating these complementary approaches is therefore essential for assessing how husbandry practices affect the final product. Despite this, the eating quality of chicken meat across different husbandry practices is predominantly assessed using physicochemical measurements alone, whereas extensive and systematic human consumer studies remain scarce. Consequently, the available consumer evidence is limited and should be interpreted with caution.

Together, these considerations highlight the complexity of balancing ethical, environmental and economic priorities in poultry production. In practice, end-products reflect combinations of multiple extensification factors, yet how these system-level profiles translate into consumer-relevant eating quality remains poorly understood.

This study was conducted within the European Union-funded *mEATquality* project, which investigates how extensification factors can be applied and optimized across housing systems for broiler chickens, one of the most intensively kept livestock species. Chicken breast meat was obtained from eight production concepts, representing different combinations of four factors (genetics, diet, space allowance, and environmental enrichment) across higher-welfare non-organic and organic systems. These concepts were implemented in the Netherlands and Germany, which were selected within the project consortium due to their established higher-welfare and organic broiler production systems. Physicochemical measurements were combined with trained sensory assessment and consumer testing, both conducted in Denmark, to examine how system-level combinations of these factors shape the eating quality of chicken breast meat. Production concepts were interpreted as integrated production profiles, where multiple husbandry factors co-vary, allowing evaluation of system-level outcomes and their relevance to consumer-perceived quality.

## Materials and methods

### Husbandry factors and experimental design

**Fig. 1:**
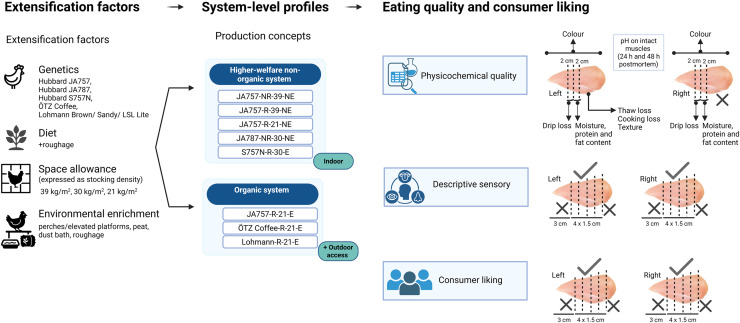
**Schematic overview of extensification factors, system-level profiles of production concepts, and assessment of eating quality and consumer liking.** Sampling locations on left and right breast fillets are shown for physicochemical quality, descriptive sensory analysis and consumer evaluation. Created in BioRender.

A detailed overview of the production concepts is provided in Table 1 and the experimental design, including eating quality and consumer liking assessments, is illustrated in Fig. 1.

**Table 1:** Overview of the eight production concepts and their associated extensification factors (genetics, diet, space allowance, environmental enrichment) across different housing systems and their inclusion in physicochemical quality, descriptive sensory and consumer liking.

Production concepts	Genetics	Diet	Space allowance	Environmental enrichment	Housing system	Physicochemical quality	Descriptive sensory	Consumer liking
JA757-NR-39-NE	Hubbard JA757	Standard *Beter-Leven* (Better Life), no roughage supplemented	39 kg/m² (high stocking density)	None	Higher welfare-non organic	*n* = 40	*n* = 20	—
JA757-R-39-NE	Hubbard JA757	Standard *Beter-Leven* (Better Life), roughage supplemented	39 kg/m² (high stocking density)	None	Higher welfare-non organic	*n* = 40	*n* = 20	*n* = 76
JA757-R-21-NE	Hubbard JA757	Standard *Beter-Leven* (Better Life), roughage supplemented	21 kg/m² (low stocking density)	None	Higher welfare-non organic	*n* = 40	*n* = 20	—
JA787-NR-30-NE	Hubbard JA787	Standard *Beter-Leven* (Better life), no roughage supplemented	30 kg/m² (medium stocking density)	None	Higher welfare-non organic	*n* = 40	*n* = 20	—
S757N-R-30-E	Hubbard S757N	Standard *Beter-Leven* (Better life), roughage supplemented	30 kg/m² (medium stocking density)	Enriched with perches, peat, dust bath and roughage	Higher welfare-non organic	*n* = 40	*n* = 20	*n* = 76
JA757-R-21-E	Hubbard JA757	Standard organic (EU) diet	21 kg/m² (low stocking density)	Enriched with perches/elevated platforms, roughage and outdoor area	Organic	*n* = 25	*n* = 20	*n* = 76
ÖTZ Coffee-R-21-E	Dual-purpose ÖTZ Coffee	Standard organic (EU) diet	21 kg/m² (low stocking density)	Enriched with perches/elevated platforms, roughage and outdoor area	Organic	*n* = 25	*n* = 20	—
Lohmann-R-21-E	Male layer Lohmann Brown, Lohmann Sandy, Lohmann LSL Lite	Standard organic (EU) diet	21 kg/m² (low stocking density)	Enriched with perches/elevated platforms, roughage and outdoor area	Organic	*n* = 25	*n* = 40	—

Production concepts are coded as: genetics; diet; space allowance; environmental enrichment. Genetics: (JA757) Hubbard JA757; (JA787) Hubbard JA787; (S757N) Hubbard S757N; (ÖTZ Coffee) Dual-purpose ÖTZ Coffee; (Lohmann) Male layer Lohmann Brown, Lohmann Sandy and Lohmann LSL Lite. Diet: (NR) No roughage; (R) Roughage supplemented. Space allowance: 21, 30, 39 kg/m². Environmental enrichment: (NE) Non-enriched; (E) Enriched. Housing systems: higher-welfare non-organic or organic. The n values in physicochemical quality and descriptive sensory columns refer to the number of broilers sampled per production concept, while n values in the consumer liking column indicate the number of consumer evaluations.

Samples from higher welfare non-organic systems, originated from the Netherlands, where all birds were raised indoors. Genotypes included Hubbard JA757 (slower-growing, slaughter age 49 days), Hubbard JA787 (faster-growing, slaughter age 42 days) and Hubbard S757N (naked-neck used for Label Rouge production; slower-growing, slaughter age 63 days) (EFSA, 2023). Diets consisted of a commercially available so-called four-phase program, as applied for slower-growing breeds under the Dutch *Beter Leven* (Better Life) concept, a one-star welfare label, provided either without (NR) or with roughage supplementation (R). Birds were reared indoors at high (39 kg/m²), medium (30 kg/m²) or low (21 kg/m²) stocking densities (Marchewka et al., 2023). Environmental enrichment, when present, included an adjustable-height barrier perch, a peat dust bath (1 × 3.13 m) and lucerne bales. Wood shavings (1.5 kg/m² per pen) were used as bedding outside the dust bath area. Concepts JA757-NR-39-NE, JA757-R-39-NE and JA757-R-21-NE were each assigned to four replicate pens. Concepts JA787-NR-30-NE and S757N-R-30-E were each assigned to five replicate pens. Birds were processed at a target live weight of ∼2.2 kg. Husbandry conditions were described in detail previously (Yigitturk et al., 2025).

Samples from organic systems, originating from five farms in Germany, were raised under the EU Organic Regulation 2018/848. These included three genetic breeds raised under standard organic environmental enrichment: Hubbard JA757 (slower-growing, slaughter ages 55, 56 and 64 days; two farms), ÖTZ Coffee (dual-purpose; slaughter ages 115 and 136 days; one farm) and Lohmann (male-layer strains; Lohmann Brown [LB], Lohmann Sandy [LS] and Lohmann LSL Lite [LSL]; slaughter age 83 days; two farms). Stocking density was 21 kg/m², and all birds had access to perches and/or elevated platforms, roughage and outdoor areas. Each concept comprised four replicates, obtained from different farms, batches, pens and genetic lines. In the Lohmann-R-21-E, LB and LS strains were housed in the same pen but sampled separately and treated as independent replicates. Three Lohmann male-layer strains were included to compensate for the limited sample sizes from individual strains. In contrast to the controlled experimental facility in the Netherlands, the German part of the study was conducted under commercial organic farm conditions.

### Sample collection

Only male broilers were analysed. Birds from Dutch production concepts were transported (∼35 min) to a processing facility in Diessen, The Netherlands. Birds were manually head-only electrically stunned (240 mA for 6 s), water scalded (60 °C for 90 s), defeathered and air chilled (∼1 °C, 24 h) under standard commercial processing conditions. Random samples were taken per concept, with 40 birds selected for physicochemical analyses and 50 birds for sensory and consumer testing, balanced across replicate pens.

Organic birds from five German farms were transported to regional slaughter plants (5 min to 6 h transport). For the longest transport (6 h), birds were transported in vehicles equipped with ventilation. All transports were carried out in accordance with the general welfare requirements during transport as set out in EU Council Regulation (EC) No 1/2005. Birds from the JA757-R-21-E were stunned using a multi-phase CO₂ gas mixture (O₂ gradually reduced from 19 % to 0 % and CO₂ increased from 27 % to 88 %), scalded in water (52.4 °C, 5 min) and air chilled (∼1 °C, 24 h). Birds from the ÖTZ Coffee-R-21-E were electrically stunned (50 Hz, 120 V, 240 A), water scalded (63 °C for 35 s) and air chilled (∼1 °C, 24 h). Birds from the Lohmann-R-21-E were electrically stunned (120 mA, 90–100 V), water scalded (63 °C for 30 s) and air chilled (∼1 °C, 24 h). For physicochemical analyses, 25 birds per concept were randomly selected. Sampling was structured to ensure representative coverage of the farm-batch-pen-genetic line hierarchy within each concept. For sensory and consumer testing, 40 birds per concept were randomly selected for JA757-R-21-E and Lohmann-R-21-E and 20 birds for ÖTZ Coffee-R-21-E (due to limited sample availability), following the same hierarchical sampling structure.

An overview of the farm, batch, pen and genetic-line structure of each production concept, including sample allocation for physicochemical analyses and sensory and consumer evaluations, is provided in Supplementary Fig. 1.

After 24 h postmortem, carcasses were vacuum-packaged. Those destined for physicochemical analyses were transported under refrigeration (4 °C) to Wageningen University (The Netherlands), while those for sensory and consumer evaluation were frozen at −18 °C and transported to the Danish Technological Institute (Taastrup, Denmark) and kept frozen (3-5 months) until analyses.

### Physicochemical quality

#### Moisture, Protein and Fat Content

At 48 h postmortem, the carcasses were dissected and both pectoralis major muscles (breast fillets) were removed for subsequent analyses. A 2 cm mid-section (steak) was cut from the left and right pectoralis major and homogenized for analysis. Moisture and protein were analysed on all available samples. For fat analysis, three broilers were selected per experimental unit. Within the Lohmann concept, three genetic strains (LB, LS and LSL) were represented and six birds from each strain were analysed (*n* = 18 in total for the Lohmann concept). This approach ensured balanced representation of all Lohmann genetic strains while maintaining consistency in sampling across other experimental units.

Moisture was determined according to AOAC Official Method 950.46, protein according to AOAC Official Method 992.15 and intramuscular fat according to AOAC Official Method 991.36, with analytical procedures as described previously (Yigitturk et al., 2025). All analyses were performed in duplicate.

#### pH and Water-holding Capacity

The breast fillet pH was measured in triplicate at 24 h and 48 h postmortem, and the average value was reported. Measurements were taken using a portable meat pH meter equipped with an insertion glass electrode (HI99163, Hanna Instruments Nederland). The electrode was calibrated with pH 4.01 and pH 7.01 buffer solutions before use, and calibration was re-checked after every ∼10 birds. The 48 h postmortem measurements were recorded to reflect the physiological condition of the muscle at the time of downstream analyses.

Water-holding capacity (WHC) was assessed through drip, thaw and cooking loss. For drip loss, a 2 cm steak sample was taken below the mid-point of each left and right *pectoralis major* (48 h postmortem) using the EZ-drip method (Correa et al., 2007). Cylindrical cores (25 mm) were excised from both breast steak samples, providing technical duplicates per bird. The cores were weighed and stored vertically at 4 °C for 24 h. Drip loss (%) was then calculated as the proportion of exuded fluid relative to the weight of the core samples. For thaw loss, the left *pectoralis major* was halved, frozen (−20 °C), thawed (24 h at 4 °C) and weighed before freezing and after thawing. Thaw loss (%) was calculated as the proportional weight loss. Cooking loss was then determined on the same halved left muscle by cooking vacuum-packaged samples in a water bath (76 °C) to an internal temperature of 72 °C, cooling and reweighing. Cooking loss (%) was calculated relative to pre-cooking weight. Thaw and cooking loss were measured on a single halved left fillet per bird.

#### Texture

The texture analysis employed the blunt Meullenet-Owens razor shear (BMORS) method (Lee et al., 2008). Cooked and refrigerated chicken samples, derived from the same portions used for thaw and cooking loss, underwent shear by a blunt blade (height 24.0 mm; width 8.0 mm; thickness 0.5 mm) affixed to a texture analyser (Model TA.XT plus, Stable Micro Systems) with a 5 kg load cell. Shearing was performed at six predetermined positions, oriented perpendicular to the muscle fibre. Both maximum shear force (N) and shear energy (N·mm) were recorded. For texture profile analysis (TPA), two cylindrical cores (25 mm diameter, 20 mm height, or 15 mm when sample thickness was insufficient) were prepared from each cooked sample, with excess trimmed from the top and bottom. Samples were then compressed 50 % of their original height with a 50 mm diameter cylindrical aluminium probe (P50, Stable Micro Systems) attached to the texture analyser equipped with a 30 kg load cell. The TPA results are reported as: hardness (N; maximum force on first compression; matrix stiffness), chewiness (N; mechanical work per chew), springiness (%; height recovery; viscoelastic rebound), cohesiveness (%; work of second compression relative to the first), and resilience (%; instantaneous elastic recovery) (Paredes et al., 2022).

#### Colour

Instrumental colour was measured using the electronic eye imaging system (IRIS VA400, Alpha M.O.S., France) under top lighting with a 16 mm aperture, calibrated with a 24-colour checker plate. Both the left and right whole *pectoralis major* fillets from each bird were positioned on a white background and imaged separately, with the two measurements considered as technical replicates. Images were analysed in Alphasoft (v14.0) to extract CIE parameters L, a* and b* values. Measurements were averaged per bird. A standard pink colour tile (Konika Minolta, Tokyo, Japan) was measured as a control at the start of each analysis session to verify consistency across measurement sessions. The total colour difference (ΔE) was derived from L*, a* and b* values using the standard CIE formula (King et al., 2023).

### Descriptive sensory analysis

The descriptive sensory analysis was performed following the protocol detailed previously (Siebenmorgen et al., 2025). The sensory descriptive analysis followed “Generic sensory descriptive analysis” (Murray et al., 2001) in an ISO 8589-compliant, accredited laboratory (DTI, Copenhagen). Briefly, salted carcasses (2 g/kg) were roasted in a combi oven (Electrolux Air-O-Stream, 175 °C) to an internal temperature of 70 ± 3 °C. Breast fillets were cut into uniform pieces (4 × 1.5 cm) and served warm on coded plates. The analysis utilized a pre-screened, accredited sensory panel (DANAK 05-0392) of eight experienced assessors (six female, mean age 56 years, sensory experience 2–15 years). The panel developed a vocabulary of 15 sensory attributes (Table 2) during initial training sessions (2 × 3 h) and participated in weekly scale alignment calibrations throughout the three-week study. Following established standards (e.g., ISO 4121), panellists evaluated coded samples in a randomized block design. Attributes were rated on a 15 cm unstructured line scale using RedJade® software (v5.1.1). Standard palate cleansers (water, crisp bread and cucumber) were provided between samples. The study was conducted in accordance with the ethical guidelines of the Danish Ministry of Higher Education and Science and the Danish Council on Ethics, as implemented by the Danish Technological Institute (DTI).

**Table 2:** Definitions and scale range of the sensory characteristics (in bold) and attributes used to evaluate the chicken breast fillet meat.

Attribute	Scale (0-15)	Definition
**Appearance**		
Colour	Light - dark	Colour of the meat
Blood spots	None - many	Red blood spots in the meat
**Odor**		
Boiled chicken	Little - much	Intensity of the odour associated with boiled chicken meat
Sweet	None - much	Intensity of sweet odour
**Flavour/taste**		
Boiled chicken	Little - much	Intensity of the flavour associated with boiled chicken meat
Sour	None - much	Intensity of sour taste
Sweet	None - much	Intensity of sweet taste
Metallic	None - much	Intensity of iron/blood flavour
**Texture**		
Firmness at first bite	Little - much	How much force is required to bite the meat with molars at the first bite
Juiciness	Little - much	Intensity of juiciness after five chews
Tenderness	Little - much	Ease with which the meat is broken down during chewing
Stringiness	None - much	Degree of how the meat separates into fibres while chewing
Crumbling	None - much	Degree of how the meat breaks into small fine pieces while chewing
**Aftertaste**		
Bitter	None - much	Intensity of a bitter taste after the sample had been spit out

### Consumer liking test and procedure

Selected samples were prepared, cooked and served using the same procedure as for the descriptive sensory evaluation. Three concepts representing distinct sensory profiles (JA757-NR-39-NE, JA757-R-21-E, and S757N-R-30-E) were selected. The selection of concepts was also determined by the limited availability of Lohmann-R-21-E and ÖTZ Coffee-R-21-E samples, which precluded their inclusion in the consumer test.

Seventy-six adults (35 females, age 18–65) were recruited from the DTI Aarhus consumer database (N > 5000), based on the criteria: age 18–65 years, consumption of chicken at least every two months, and self-reported liking of chicken. The study was conducted over two days in the ISO 8589-compliant sensory laboratory at DTI. After a brief introduction, the consumers were seated individually in sensory booths equipped with a computer (data collection RedJade® v5.1.1) and were encouraged to use water and neutral crackers after each sample.

Each consumer evaluated each concept twice, first in a blinded format and then in an informed format; only data from the blinded format are presented in this paper as the data from the informed format is out of scope for this paper and will be published elsewhere. For each sample, the consumers rated *overall liking, liking of appearance, odour, taste, tenderness, juiciness*, and *texture* on a 9-point hedonic scale (1 = “dislike extremely,” 9 = “like extremely”). Sample order was randomized in alignment with the sensory panel procedure. All participants received a gift voucher.

Written informed consent was obtained, and all procedures complied with Danish ethical and data protection regulations for sensory studies with healthy adults.

### Data analysis

Data were aggregated at the experimental-unit level for each system. For the Dutch trials, each production concept contained a single farm and batch, and pens were the only source of within-concept variation. Pen means were therefore used as the experimental unit. For the German trials, production concepts were represented across multiple farms, batches and genetic lines within a genotype. In these cases, data were first summarized at the farm-batch-pen-genetic line level to preserve the hierarchical design, and these aggregated means were used as the experimental units for statistical analysis. An overview of the resulting experimental units used for statistical analyses is provided in Supplementary Fig. 1.

Physicochemical quality data were analysed using *R* (version 4.4.1). For each trait, a linear model of the form *y = μ + production concept + ε* was fitted to the aggregated experimental units described above, with production concept treated as a fixed effect. When a significant concept effect was detected, pairwise comparisons were obtained using Tukey’s HSD adjustment.

To integrate physicochemical quality traits with descriptive sensory attributes, a partial least squares regression (PLSR) analysis was performed at the experimental unit level. Data were mean-centred and auto-scaled prior to analysis. Model interpretation focused on score and loading plots rather than predictive performance. The PLSR analysis was performed in *R* (version 4.4.1) using the *mixOmics* package.

A principal component analysis (PCA) on mean sensory data was applied to visualize the relationship between the descriptive analysis attributes and the chicken breast samples from different production concepts (RedJade® software (v5.1.1)). The model was auto-scaled and full cross validated. To further elucidate the relationship between the descriptive analysis attributes and the samples, ANOVA models and subsequently LSD denotations were developed for post-hoc analysis.

To test differences in consumers overall liking and liking for the modalities (appearance, taste, juiciness, tenderness and texture) for the chicken breast fillet samples mixed model ANOVAs were also performed with samples as main effects and subjects as random effects (XLSTAT, Version 2024.2.2, addinsoft SARL, Paris, France).

For all data analysed, a *P*-value of less than 0.05 was considered statistically significant.

## Results

### Physicochemical quality

Physicochemical quality traits differed significantly among production concepts, as shown by univariate analyses (Table 3, Table 4, Table 5, Table 6). A PCA was used to integrate these traits and to visualise their multivariate relationships (Supplementary Fig. 2).

**Table 3:** Breast meat compositions across production concepts.

Production concepts	Moisture (%)	Protein (wet basis, %)	Fat (wet basis, %)	Protein-to-fat ratio
JA757-NR-39-NE	74.2^b^ ± 0.33 (*n* = 40)	23.8^abc^ ± 0.33 (*n* = 40)	0.52^a^ ± 0.08 (*n* = 12)	48.0^b^ ± 5.97 (*n* = 12)
JA757-R-39-NE	73.9^b^ ± 0.17 (*n* = 40)	23.9^abc^ ± 0.18 (*n* = 40)	0.46^ab^ ± 0.06 (*n* = 12)	60.7^b^ ± 7.65 (*n* = 12)
JA757-R-21-NE	73.8^b^ ± 0.20 (*n* = 40)	23.8^abc^ ± 0.28 (*n* = 40)	0.42^ab^ ± 0.10 (*n* = 12)	68.7^b^ ± 12.52 (*n* = 12)
JA787-NR-30-NE	74.7^b^ ± 0.07 (*n* = 40)	23.5^bc^ ± 0.29 (*n* = 40)	0.53^a^ ± 0.20 (*n* = 15)	68.7^b^ ± 29.02 (*n* = 12)
S757N-R-30-E	74.0^b^ ± 0.13 (*n* = 40)	24.3^a^ ± 0.36 (*n* = 40)	0.17^c^ ± 0.02 (*n* = 15)	197.7^a^ ± 45.17 (*n* = 12)
JA757-R-21-E	74.2^b^ ± 0.81 (*n* = 25)	23.0 ^cd^ ± 0.48 (*n* = 25)	0.53^a^ ± 0.11 (*n* = 12)	46.3^b^ ± 11.32 (*n* = 12)
ÖTZ Coffee-R-21-E	74.3^b^ ± 0.41 (*n* = 25)	24.1^ab^ ± 0.44 (*n* = 25)	0.35^abc^ ± 0.07 (*n* = 12)	70.7^b^ ± 13.08 (*n* = 12)
Lohmann-R-21-E	75.6^a^ ± 0.57 (*n* = 25)	22.4^d^ ± 0.51 (*n* = 25)	0.24^bc^ ± 0.04 (*n* = 18)	97.2^b^ ± 13.64 (*n* = 12)
*P*-value	< 0.001***	< 0.001***	< 0.001***	< 0.001***

Mean ± standard deviation (n refers to the number of birds analysed per production concept). Statistical analysis was performed on aggregated experimental units, defined according to the trial structure (see *Data Analysis*). Production concept effects were assessed using one-way linear models (ANOVA) followed by Tukey’s HSD post hoc test. Different superscript letters (a-d) within a column indicate statistically significant differences (***P < 0.001); values sharing no common letter differ at least *P* < 0.05.

**Table 4:** Postmortem pH values and water-holding capacity of breast meat fillets across production concepts.

Production concepts	pH postmortem 24 h	pH postmortem 48 h	Drip loss (%)	Thaw loss (%)	Cooking loss (%)
JA757-NR-39-NE	5.78^bc^ ± 0.01 (*n* = 40)	5.84^bc^ ± 0.04 (*n* = 40)	0.81^b^ ± 0.13 (*n* = 40)	10.86^ab^ ± 0.60 (*n* = 40)	12.75^a^ ± 0.44 (*n* = 40)
JA757-R-39-NE	5.86^ab^ ± 0.05 (*n* = 40)	5.89^ab^ ± 0.04 (*n* = 40)	0.75^b^ ± 0.14 (*n* = 40)	10.34^ab^ ± 1.27 (*n* = 40)	11.69^ab^ ± 0.49 (*n* = 40)
JA757-R-21-NE	5.80^abc^ ± 0.02 (*n* = 40)	5.86^abc^ ± 0.04 (*n* = 40)	0.72^b^ ± 0.12 (*n* = 40)	11.30^a^ ± 0.36 (*n* = 40)	12.41^ab^ ± 0.74 (*n* = 40)
JA787-NR-30-NE	5.89^ab^ ± 0.04 (*n* = 40)	5.90^ab^ ± 0.02 (*n* = 40)	0.64^b^ ± 0.14 (*n* = 40)	10.30^ab^ ± 1.21 (*n* = 40)	12.10^ab^ ± 0.38 (*n* = 40)
S757N-R-30-E	5.93^a^ ± 0.06 (*n* = 40)	5.89^ab^ ± 0.07 (*n* = 40)	0.42^b^ ± 0.05 (*n* = 40)	8.58^b^ ± 0.80 (*n* = 40)	8.98^c^ ± 0.40 (*n* = 40)
JA757-R-21-E	5.67^c^ ± 0.09 (*n* = 25)	5.83^bc^ ± 0.05 (*n* = 25)	1.93^a^ ± 0.89 (*n* = 25)	9.89^ab^ ± 0.99 (*n* = 25)	12.13^ab^ ± 1.52 (*n* = 25)
ÖTZ Coffee-R-21-E	5.79^bc^ ± 0.11 (*n* = 25)	5.76^c^ ± 0.11 (*n* = 25)	0.64^b^ ± 0.21 (*n* = 25)	5.25^c^ ± 2.19 (*n* = 25)	10.51^bc^ ± 1.02 (*n* = 25)
Lohmann-R-21-E	5.93^a^ ± 0.04 (*n* = 25)	5.99^a^ ± 0.05 (*n* = 25)	0.80^b^ ± 0.21 (*n* = 25)	9.22^ab^ ± 1.38 (*n* = 25)	10.96^ab^ ± 1.37 (*n* = 25)
*P*-value	< 0.001***	< 0.001***	< 0.001***	< 0.001***	< 0.001***

Mean ± standard deviation (n refers to the number of birds analysed per production concept). Statistical analysis was performed on aggregated experimental units, defined according to the trial structure (see *Data Analysis*). Production concept effects were assessed using one-way linear models (ANOVA) followed by Tukey’s HSD post hoc test. Different superscript letters (a-c) within a column indicate statistically significant differences (***P < 0.001); values sharing no common letter differ at least *P* < 0.05.

**Table 5:** Shear force and texture profile analysis results of breast meat fillets across production concepts.

Production concepts	Shear force (N)	Total shear energy (N.mm)	Hardness (N)	Chewiness (N)	Springiness (%)	Cohesiveness (%)	Resilience (%)
JA757-NR-39-NE	10.44^ab^ ± 0.60 (*n* = 40)	101.04^a^ ± 8.65 (*n* = 40)	77.75^ab^ ± 4.33 (*n* = 40)	9.95^bc^ ± 0.51 (*n* = 40)	24.94^bc^ ± 0.66 (*n* = 40)	51.48^b^ ± 0.90 (*n* = 40)	20.99^bc^ ± 0.80 (*n* = 40)
JA757-R-39-NE	9.58^abcd^ ± 0.29 (*n* = 40)	95.59^a^ ± 6.02 (*n* = 40)	73.45^abc^ ± 2.70 (*n* = 40)	9.33^bc^ ± 0.26 (*n* = 40)	25.13^bc^ ± 0.67 (*n* = 40)	50.29^b^ ± 1.41 (*n* = 40)	20.63^c^ ± 0.66 (*n* = 40)
JA757-R-21-NE	10.11^abc^ ± 0.98 (*n* = 40)	97.90^a^ ± 9.53 (*n* = 40)	69.29^bc^ ± 4.91 (*n* = 40)	9.86^bc^ ± 0.89 (*n* = 40)	28.29^ab^ ± 0.90 (*n* = 40)	50.22^b^ ± 1.44 (*n* = 40)	20.72^c^ ± 0.59 (*n* = 40)
JA787-NR-30-NE	8.54^d^ ± 0.28 (*n* = 40)	86.55^a^ ± 3.09 (*n* = 40)	60.09^c^ ± 3.38 (*n* = 40)	8.71^c^ ± 0.80 (*n* = 40)	29.82^a^ ± 1.75 (*n* = 40)	48.32^b^ ± 0.80 (*n* = 40)	20.47^c^ ± 0.48 (*n* = 40)
S757N-R-30-E	10.90^a^ ± 0.69 (*n* = 40)	95.61^a^ ± 11.29 (*n* = 40)	65.15^bc^ ± 5.70 (*n* = 40)	8.12^c^ ± 0.49 (*n* = 40)	24.55^c^ ± 1.67 (*n* = 40)	51.10^b^ ± 1.90 (*n* = 40)	22.04^bc^ ± 1.17 (*n* = 40)
JA757-R-21-E	8.88^bcd^ ± 0.75 (*n* = 25)	84.55^a^ ± 7.02 (*n* = 25)	78.90^ab^ ± 7.64 (*n* = 25)	10.31^bc^ ± 1.32 (*n* = 25)	25.39^bc^ ± 1.39 (*n* = 25)	51.25^b^ ± 1.05 (*n* = 25)	20.66^c^ ± 0.45 (*n* = 25)
ÖTZ Coffee-R-21-E	10.28^ab^ ± 0.58 (*n* = 25)	94.76^a^ ± 9.98 (*n* = 25)	85.84^a^ ± 14.01 (*n* = 25)	11.42^b^ ± 2.14 (*n* = 25)	25.39^bc^ ± 1.46 (*n* = 25)	52.79^b^ ± 4.07 (*n* = 25)	23.55^b^ ± 2.35 (*n* = 25)
Lohmann-R-21-E	8.61 ^cd^ ± 1.06 (*n* = 25)	53.79^b^ ± 11.11 (*n* = 25)	85.70^a^ ± 1.30 (*n* = 25)	13.88^a^ ± 0.73 (*n* = 25)	26.42^bc^ ± 2.35 (*n* = 25)	60.65^a^ ± 2.96 (*n* = 25)	26.36^a^ ± 1.83 (*n* = 25)
*P*-value	< 0.001***	< 0.001***	< 0.001***	< 0.001***	< 0.001***	< 0.001***	< 0.001***

Mean ± standard deviation (n refers to the number of birds analysed per production concept). Statistical analysis was performed on aggregated experimental units, defined according to the trial structure (see *Data Analysis*). Production concept effects were assessed using one-way linear models (ANOVA) followed by Tukey’s HSD post hoc test. Different superscript letters (a-d) within a column indicate statistically significant differences (***P < 0.001); values sharing no common letter differ at least *P* < 0.05. Peak shear force and shear energy were derived from the Blunt Meullenet-Owens Razor Shear (BMORS). Texture profile parameters (hardness, chewiness, springiness, cohesiveness, resilience) were derived from performing texture profile analysis.

**Table 6:** Colour parameters of breast meat across production concepts.

Production concepts	Lightness, L*	Redness, a*	Yellowness, b*
JA757-NR-39-NE	66.27^bcd^ ± 0.93 (*n* = 40)	8.02^bc^ ± 0.51 (*n* = 40)	18.71^a^ ± 0.29 (*n* = 40)
JA757-R-39-NE	65.23 ^cd^ ± 0.70 (*n* = 40)	8.40^bc^ ± 0.20 (*n* = 40)	18.45^ab^ ± 0.85 (*n* = 40)
JA757-R-21-NE	65.94^bcd^ ± 0.58 (*n* = 40)	8.10^bc^ ± 0.20 (*n* = 40)	18.87^a^ ± 0.41 (*n* = 40)
JA787-NR-30-NE	58.53^e^ ± 1.05 (*n* = 40)	10.85^a^ ± 0.36 (*n* = 40)	16.28^c^ ± 0.41 (*n* = 40)
S757N-R-30-E	64.99^d^ ± 1.36 (*n* = 40)	8.71^bc^ ± 0.47 (*n* = 40)	16.98^bc^ ± 0.49 (*n* = 40)
JA757-R-21-E	68.58^ab^ ± 1.21 (*n* = 25)	7.96^c^ ± 0.75 (*n* = 25)	19.07^a^ ± 0.89 (*n* = 25)
ÖTZ Coffee-R-21-E	68.26^abc^ ± 2.83 (*n* = 25)	10.16^ab^ ± 2.45 (*n* = 25)	15.65^c^ ± 0.94 (*n* = 25)
Lohmann-R-21-E	69.84^a^ ± 1.63 (*n* = 25)	8.58^bc^ ± 0.18 (*n* = 25)	19.01^a^ ± 1.18 (*n* = 25)
*P*-value	< 0.001***	< 0.001***	< 0.001***

Mean ± standard deviation (n refers to the number of birds analysed per production concept). Statistical analysis was performed on aggregated experimental units, defined according to the trial structure (see *Data Analysis*). Production concept effects were assessed using one-way linear models (ANOVA) followed by Tukey’s HSD post hoc test. Different superscript letters (a-e) within a column indicate statistically significant differences (***P < 0.001); values sharing no common letter differ at least *P* < 0.05.

#### Moisture, Protein and Fat Content

Breast meat compositional traits across production concepts are presented in Table 3. Moisture content was highest in Lohmann-R-21-E (83 days, second-oldest birds in the study), exceeding all other concepts by 0.9-1.8 %.

The protein content was highest in S757N-R-30-E (63 days), with ÖTZ Coffee-R-21-E (115-136 days, oldest birds) also among the upper range. Organic JA757-R-21-E and Lohmann-R-21-E were at the lower end, although the organic JA757 overlapped statistically with the higher-welfare non-organic JA757 concepts (JA757-NR-39-NE, JA757-R-39-NE, JA757-R-21-NE). The fat content displayed the opposite pattern. The highest values were found in JA787-NR-30-NE, JA757-R-21-E and JA757-NR-39-NE, while S757N-R-30-E meat was the leanest. Consequently, S757N-R-30-E showed the highest protein-to-fat ratio, significantly exceeding all other concepts by two-to-fourfold.

#### pH and Water-holding Capacity

Postmortem pH values and WHC of breast meat across production concepts are presented in Table 4. Postmortem pH values ranged from 5.7 to 6.0, with similar patterns observed at both 24 h and 48 h time points. All pH values remained within the normal range for quality chicken breast (Beauclercq et al., 2022).

S757N-R-30-E combined high pH with drip loss and cooking loss at the lower end of the range, alongside one of the lowest thaw losses. JA757-R-21-E had a pH at the lower end of the range and exhibited more than fourfold higher drip loss, along with higher thaw and cooking losses. Consistent with these findings, the PCA (Supplementary Fig. 2) showed drip, thaw and cooking losses clustering together and loading opposite to postmortem pH 24. In contrast, ÖTZ Coffee-R-21-E showed pH values at the lower side of the range together with the lowest thaw loss and lower cooking loss, likely reflecting its older slaughter age (115 days).

#### Mechanical Texture Characteristics

Mechanical texture characteristics of breast meat across production concepts are presented in Table 5. Shear force ranged from 8.54 to 10.90 N, with S757N-R-30-E showing the highest value, while several concepts (including JA757-R-21-E, Lohmann-R-21-E and JA787-NR-30-NE) fell at the lower end of the range. Total shear energy was similar across most concepts (85-101 N.mm), except for Lohmann-R-21-E, which had a significantly lower value, approximately half that of the other concepts.

The PCA (Supplementary Fig. 2) indicated an inverse relationship between shear-based toughness (shear force and energy) and compression-based firmness (hardness, chewiness, cohesiveness and resilience), reflecting distinct textural dimensions that were separated from water-holding-related traits. Hardness was highest in the two oldest groups, ÖTZ Coffee-R-21-E (115-136 days) and Lohmann-R-21-E (83 days), which differed significantly from S757N-R-30-E and JA787-NR-30-NE. Chewiness followed a similar pattern with Lohmann-R-21-E and ÖTZ Coffee-R-21-E exceeding S757N-R-30-E and JA787-NR-30-NE. Springiness was highest in JA787-NR-30-NE, underscoring its elastic character, while S757N-R-30-E was less bouncy. Cohesiveness and resilience were highest in Lohmann-R-21-E and the lowest in JA787-NR-30-NE.

No consistent differences were observed between JA757 concepts with or without dietary fibre supplementation (JA757-R-39-NE vs. JA757-NR-39-NE) or between high- and low-density indoor systems (JA757-R-39-NE vs. JA757-R-21-NE). Likewise, placing the same genotype (JA757) in an organic system (JA757-R-21-E) did not significantly alter texture.

#### Colour

Colour parameters of breast meat across production concepts are presented in Table 6. Lightness (L*) was significantly higher in Lohmann-R-21-E, JA757-R-21-E and ÖTZ Coffee-R-21-E, all from the organic system, indicating paler meats. In contrast, JA787-NR-30-NE showed the lowest L* value, consistent with its darker appearance observed in the sensory colour evaluation (see Table 7a). Redness (a*) was highest in JA787-NR-30-NE and ÖTZ Coffee-R-21-E, while the remaining concepts, including JA757-R-21-E, had lower and similar values. Yellowness (b*) was higher in all JA757-based concepts from both systems and in Lohmann-R-21-E, but lowest in ÖTZ Coffee-R-21-E and JA787-NR-30-NE.

**Table 7:** Mean (± standard deviation) of the sensory descriptive attributes of chicken breast meat. (a) Appearance, odour, flavour and taste (b) Texture and aftertaste.

(a)								
Production concepts	Colour	Blood spots	Boiled chicken odour	Sweet odour	Boiled chicken flavour	Sour taste	Sweet taste	Metallic flavour
JA757-NR-39-NE	2.9^ab^ (±2.14)	3.3^ab^ (±0.80)	9.7 (±1.86)	6.3 (±1.12)	9.7^ab^ (±1.87)	6.3^ab^ (±2.71)	5.2 (±1.78)	4.2 (±1.72)
JA757-R-39-NE	2.6^ab^ (±1.88)	3.2^abc^ (±0.77)	9.9 (±1.62)	6.3 (±1.02)	9.6^ab^ (±1.88)	6.9^a^ (±2.60)	5.0 (±1.67)	4.0 (±1.83)
JA757-R-21-NE	2.8^ab^ (±1.81)	2.7^bc^ (±0.82)	9.8 (±1.65)	5.8 (±1.31)	9.7^a^ (±1.67)	6.5^ab^ (±2.21)	5.1 (±2.01)	4.6 (±1.80)
JA787-NR-30-NE	3.4^a^ (±2.31)	3.9^a^ (±1.44)	8.7 (±1.44)	6.1 (±1.23)	8.9^ab^ (±1.64)	5.8^ab^ (±2.12)	6.2 (±2.41)	4.4 (±2.84)
S757N-R-30-E	3.6^a^ (±2.38)	3.0^abc^ (±1.19)	8.8 (±1.44)	6.0 (±1.72)	8.8^ab^ (±1.37)	5.6^ab^ (±2.18)	6.0 (±2.35)	3.7 (±2.06)
JA757-R-21-E	2.1^b^ (±1.42)	2.1^c^ (±0.58)	8.6 (±1.21)	6.0 (±1.66)	8.8 ^ab^ (±1.51)	7.3^a^ (±2.30)	4.9 (±2.18)	4.4 (±2.05)
ÖTZ Coffee-R-21-E	3.1^ab^ (±2.02)	3.9^a^ (±1.48)	8.7 (±1.07)	5.8 (±1.70)	8.9^ab^ (±1.07)	5.7^sb^ (±2.30)	5.7 (±2.14)	4.0 (±2.73)
Lohmann-R-21-E	2.6^ab^ (±1.88)	2.7^bc^ (±0.85)	8.3 (±1.36)	5.6 (±1.69)	8.1^b^ (±1.51)	5.0^b^ (±1.49)	6.2 (±2.09)	4.1 (±2.32)

(b)								
Production concepts	Hardness at first bite	Juiciness	Tenderness	Stringiness	Crumbling	Bitter aftertaste		
JA757-NR-39-NE	4.2^b^ (±1.74)	9.4^a^ (±1.15)	10.7^a^ (±1.41)	5.7 (±2.84)	9.1 (±2.04)	4.4^b^ (±2.70)		
JA757-R-39-NE	4.1^b^ (±1.15)	8.7^ab^ (±0.94)	10.6^ab^ (±1.50)	5.5 (±2.31)	9.4 (±1.92)	4.4^b^ (±2.60)		
JA757-R-21-NE	5.0^ab^ (±1.83)	8.1^ab^ (±1.08)	9.6^abc^ (±1.38)	6.6 (±2.44)	8.9 (±1.91)	5.0^ab^ (±3.14)		
JA787-NR-30-NE	4.7^ab^ (±2.50)	6.7 ^cd^ (±1.68)	10.1^abc^ (±2.38)	6.1 (±2.71)	9.6 (±1.68)	5.0^ab^ (±2.96)		
S757N-R-30-E	6.1^a^ (±2.72)	6.2^d^ (±1.30)	8.5^c^ (±2.39)	7.1 (±2.59)	8.8 (±1.94)	4.8^ab^ (±2.80)		
JA757-R-21-E	5.3^ab^ (±2.50)	6.4 ^cd^ (±1.49)	9.4^abc^ (±2.67)	7.0 (±3.16)	9.3 (±2.32)	5.7^a^ (±3.26)		
ÖTZ Coffee-R-21-E	5.5^ab^ (±2.63)	6.4 ^cd^ (±1.52)	9.6^abc^ (±2.16)	6.0 (±2.51)	9.1 (±2.07)	4.7^ab^ (±2.63)		
Lohmann-R-21-E	5.5^ab^ (±1.92)	7.7^bc^ (±1.05)	8.9^bc^ (±1.85)	7.0 (±2.65)	8.1 (±3.02)	4.5^ab^ (±2.98)		

Different superscript letters (a-d) within a column indicate statistically significant differences between production concepts (LSD test; *P* < 0.05). Scale used: 0= light/ little/ none; 15= dark/ many/ much.

To support interpretation of the instrumental colour data, total colour differences (ΔE), derived from L*, a* and b* values, were calculated for selected concept pairs that are later shown to differ in sensory colour perception (Table 7a; JA787-NR-30-NE vs. JA757-R-21-E and S757N-R-30-E vs. JA757-R-21-E). The ΔE values confirmed large perceptual differences, with JA787-NR-30-NE vs. JA757-R-21-E (ΔE = 10.83) and S757N-R-30-E vs. JA757-R-21-E (ΔE = 4.23).

### Descriptive sensory analysis

Breast fillets from all concepts analysed for physicochemical quality were also evaluated by descriptive sensory analysis.

**Fig. 2:**
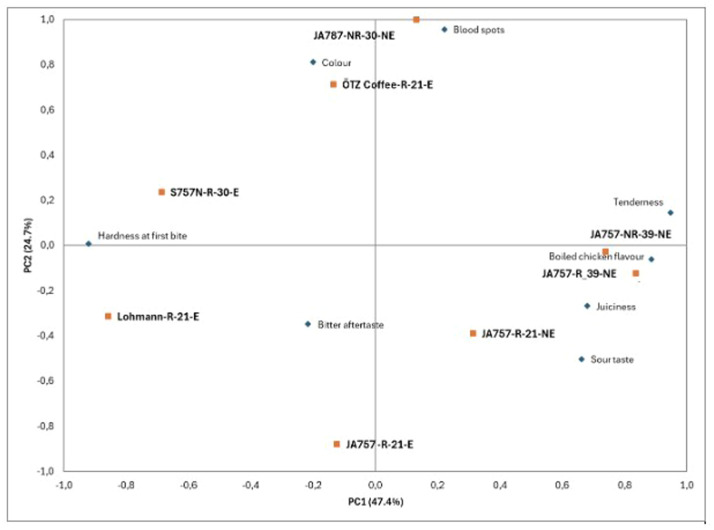
**Principal component analysis (PCA) of sensory attributes of chicken breast meat from different production concepts.** PCA biplot of factor 1 (PC1) versus factor 2 (PC2) showing sensory evaluation patterns of chicken breast fillet meat across production concepts. The data was auto-scaled and fully cross-validated.

The PCA plot shown in Fig. 2 provides an overview of the trained sensory assessors’ evaluation of chicken breast fillet samples from the eight concepts. Along PC1, the JA757 concepts from the higher-welfare non-organic system (JA757-NR-39-NE, JA757-R-39-NE, JA757-R-21-NE) projected to the positive side and co-located with tenderness, juiciness and boiled chicken flavour*.* In contrast, S757N-R-30-E and Lohmann-R-21-E projected to the negative side of PC1 and were nearest to hardness at first bite. The organic JA757-R-21-E was positioned on the negative side of both PC1 and PC2 and lay closest to bitter aftertaste (with some proximity to sour taste). Along PC2, ÖTZ Coffee-R-21-E and JA787-NR-30-NE grouped on the positive axis, aligning with colour and blood spots (appearance attributes).

Univariate tests (Table 7a, b) supported the PCA structure, showing several attributes differed significantly among concepts. The differences between the concepts were mostly related to the texture and the most consistent contrasts were observed for juiciness (Table 7b). S757N-R-30-E scored significantly lower in juiciness than the higher-welfare non-organic JA757 concepts (JA757-NR-39-NE, JA757-R-39-NE, JA757-R-21-NE), which in turn scored higher in juiciness than the organic JA757-R-21-E.

Differences in colour attributes were also noted (Table 7a). JA787-NR-30-NE and S757N-R-30-E were evaluated significantly darker (higher colour value) than JA757-R-21-E, while ÖTZ Coffee-R-21-E and JA787-NR-30-NE showed a significantly higher score for blood spots than JA757-R-21-NE, Lohmann-R-21-E and JA757-R-21-E. For flavour, the only significant contrast was boiled chicken flavour, which was scored lowest in Lohmann-R-21-E.

### Consumer liking responses

Following the physicochemical and sensory evaluations, a consumer study was conducted to assess liking scores for a subset of breast meat. Samples for the consumer study were selected to represent the most distinct sensory profiles identified in the descriptive sensory analysis, which were JA757-R-39-NE, JA757-R-21-E and S757N-R-30-E.

**Fig. 3:**
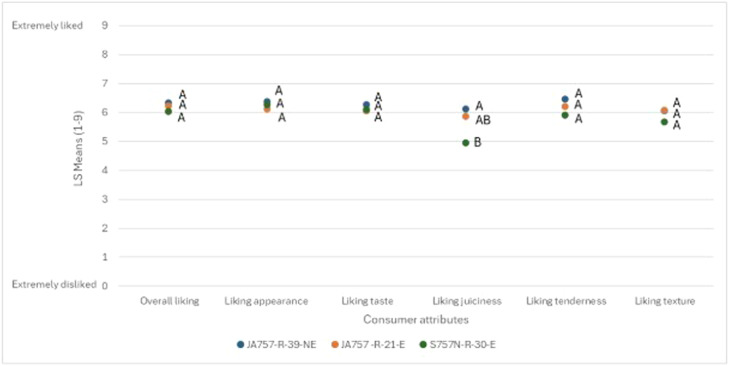
**Consumer liking of chicken breast meat from different production concepts.** The LS-means for consumer hedonic evaluations of chicken breast fillet meat across three production concepts. Different letters (*A, B*) indicate significant differences between production concepts for each attribute (Tukey HSD, *P* < 0.05). Scale used: 0= extremely disliked; 9= extremely liked.

Consumer hedonic scores for chicken breast meat from the selected concepts were rated generally similar (Fig. 3). The only significant difference was observed for *liking juiciness,* where S757N-R-30-E was rated lower than JA757-R-39-NE. No significant differences were detected for *overall liking, liking appearance, liking taste, liking tenderness* or *liking texture*. Numerically, S757N-R-30-E showed lower values for *overall liking, liking tenderness* and *liking texture*, although these differences were not statistically significant.

## Discussion

This study evaluated system-level combinations of extensification factors (genetics × diet × space allowance × environmental enrichment) to understand how they shape physicochemical quality, descriptive sensory attributes and consumer liking. Outcomes were therefore interpreted as product profiles rather than as effects of individual factors.

**Fig. 4:**
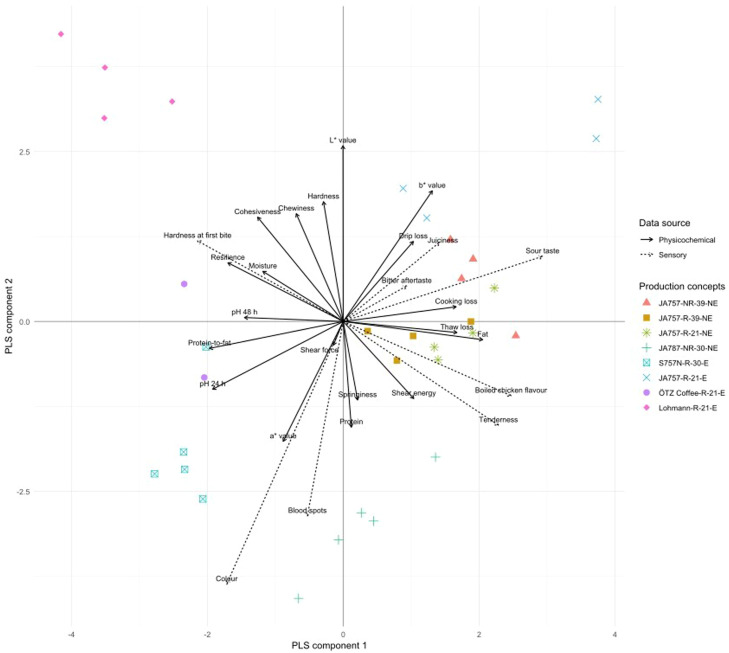
**Partial least squares regression (PLSR) biplot integrating physicochemical quality traits and descriptive sensory attributes across production concepts.** Points represent experimental units, coloured and shaped by production concept. Solid arrows indicate physicochemical variables and dashed arrows indicate sensory attributes. sensory attributes.

S757N-R-30-E produced lean, protein-dense meat (Table 3) with higher pH and one of the lowest overall water losses (∼18 %; Table 4), indicating higher WHC under moist-heat testing. Yet, it was perceived as less juicy when roasted (Fig. 3). To help explain this, the PLSR shows that sensory juiciness is associated with water-loss-related traits (drip, thaw and cooking losses), while texture-related parameters, including compression-based firmness and shear force, are oriented in a different direction in the PLSR space (Fig. 4). These findings suggest that water retention and release are modulated by textural properties, with lipid content likely further influencing oral juiciness. Although higher pH generally enhances myofibrillar protein water binding and WHC, when moisture content is similar across concepts (Table 4), juiciness can be influenced more by fat content and textural resistance. Low fat and higher firmness in S757N-R-30-E, evidenced by its higher shear force (Table 5) and higher panel-assessed hardness at first bite (Table 7b), can limit water release during mastication. This ultimately results in a drier mouthfeel (Huff-Lonergan and Lonergan, 2005; Bowker and Zhuang, 2015; Zhang et al., 2022). A deviation from the expected relationship between lower pH and poorer WHC was observed in ÖTZ Coffee-R-21-E, which, despite having one of the lowest pH values, showed the lowest overall water loss among concepts, (∼16 %; Table 4), together with higher TPA hardness (Table 5). This pattern may reflect age-related intramuscular connective-tissue maturity, through heat-stable collagen cross-links, may have increased compressive hardness and altered WHC in older birds (Listrat et al., 2016).

Shear force identified the toughest sample (S757N-R-30-E; Table 5), which also showed the lowest panel-assessed tenderness (Table 7b). However, this relationship was not entirely linear; for example, JA787-NR-30-NE had the lowest shear force but only moderate panel tenderness. Lohmann-R-21-E had the highest hardness, chewiness, cohesiveness and resilience, combined with the lowest shear energy, the highest moisture (Table 3), higher ultimate pH and moderate WHC (Table 4). This profile reflects a matrix that resists compression and rebounds efficiently, but once fractured breaks easily (Lee et al., 2008). The PLSR further supports this multidimensional interpretation, demonstrating that shear-based and compression-based texture parameters load differently in relation to sensory tenderness and juiciness. Sensory hardness at first bite aligned more closely with compression-based texture parameters (Fig. 4). Overall, these patterns suggest that shear force primarily reflects resistance to myofibrillar fracture, while TPA hardness reflects the compressive stiffness of the cooked protein network, which is strongly affected by moisture and connective-tissue cross-linking (Caine et al., 2003; Robyn et al., 2021; Bulgaru et al., 2022; Paredes et al., 2022). Tenderness is therefore best interpreted multi-dimensionally (Szczesniak, 2002).

Methodological differences should also be considered. Cooking loss and instrumental texture were measured on water-heated breast fillets (vacuum-sealed in plastic bags) to standardize heat transfer, whereas the descriptive panel and consumers evaluated oven-roasted samples to reflect consumer-relevant preparation. Because cooking method modulates moisture release, collagen solubilization, surface drying and Maillard flavour formation, absolute juiciness/firmness can shift across datasets (Liu et al., 2022; Warner et al., 2022; Roy and Bruce, 2024). For this reason, emphasis is placed on relative concept patterns rather than absolute values.

The PLSR supports the integration of instrumental and sensory colour attributes, with sensory colour and blood spots clustering together with instrumental redness (a* value) and loading opposite to instrumental lightness (L* value; Fig. 4). Total colour differences (ΔE) between sensory-differentiated concepts (JA787-NR-30-NE vs. JA757-R-21-E and S757N-R-30-E vs. JA757-R-21-E) confirmed that these contrasts exceeded human perceptual thresholds (ΔE >1), meaning that the colour differences were perceptible to the human eye (King et al., 2023). However, these differences did not translate into differences in consumer *liking appearance* for JA757-R-21-E and S757N-R-30-E (Fig. 3), indicating that while instruments and trained panellists readily detected colour variations, this was less influential for consumers than juiciness cues. While consumer purchasing decisions are significantly influenced by the visual appearance of raw meat (Altmann et al., 2022), much less is known about how the appearance of cooked meat (as tested in this study) affects consumer perception (Barbut, 2001; Roccatello et al., 2024).

In the PLSR space, the JA757-based concepts clustered together and aligned with sensory tenderness and juiciness, whereas S757N-R-30-E was clearly separated and loaded in the opposite direction of the tenderness-juiciness axis (Fig. 4). Previous studies similarly report that intermediate or modern broiler lines often exhibit higher tenderness, whereas slower-growing or older birds tend to show increased firmness (Chumngoen and Tan, 2015) and sometimes less favourable flavour profiles (Fanatico et al., 2006; Siekmann et al., 2018). However, in some markets, such as France, the stronger flavour of slower-growing genotypes (e.g., Label Rouge; represented in this study by the S757N-R-30-E) is valued by consumers, despite less tenderness (Smith et al., 2012). By contrast, Danish consumers in the present study perceived only slight differences, with only *liking of juiciness* differing significantly, even scoring S757N-R-30-E lower than JA757-R-39-NE (Fig. 3). Notably, JA757-R-39-NE combined lower first-bite hardness with higher panel-assessed tenderness and juiciness (Table 7b), which translated into higher consumer *liking of juiciness.* This consistency between PLSR outcomes and consumer responses highlights the central role of the tenderness-juiciness axis in consumer acceptance (Roccatello et al., 2024; Xu and Falsafi, 2024).

The clustering of JA757-based concepts in the PLSR space (Fig. 4) indicates that genotype was the dominant driver of the integrated sensory-physicochemical profile, with production system effects contributing secondary within-genotype variation. Within the organic JA757-R-21-E concept, the relative proximity of two units may additionally relate to differences in slaughter age (8-9 days) at a comparable target weight and/or farm-specific effects (see Materials and Methods). More generally, factors typical of organic systems, such as higher activity levels and dietary differences (e.g., pasture access) (Göransson and Lundmark Hedman, 2024), may contribute to variation in water retention and flavour perception from organic and non-organic JA757-based concepts (Table 4, Table 7) (Cartoni Mancinelli et al., 2021; Gou et al., 2021). For example, higher levels of polyunsaturated fatty acids in organic chicken meat are known to increase susceptibility to lipid oxidation, which can produce volatile compounds associated with off-flavours such as bitter aftertaste (Jayasena et al., 2013; Cartoni Mancinelli et al., 2021). Despite these contrasts, Danish consumers did not detect significant differences in liking among the organic and non-organic tested JA757 concepts (Fig. 3; JA757-R-21-E and JA757-R-39-NE). This aligns with prior reports that welfare-oriented adjustments through extensification factors (e.g., lower stocking density, provision of enrichment and dietary supplements) and alternative housing systems (higher-welfare indoor vs. organic with outdoor access) can improve welfare outcomes, but their effects on eating quality tend to be modest or inconsistent compared with the stronger effects of genotype (Baéza et al., 2022; Marchewka et al., 2023; Campbell et al., 2025).

## Conclusions

Among the extensification factors, genotype emerged as the most consistent and principal driver of the overall eating quality of chicken breast fillet meat. Differences among Danish consumers were modest and concentrated on juiciness: the higher-welfare non-organic JA757 concepts (slower-growing) were characterized by higher panel tenderness, juiciness and boiled chicken flavour, and one of these (JA757-R-39-NE) also received a higher consumer *liking juiciness* score. By contrast, S757N-R-30-E (slower-growing) produced lean, high-WHC meat that was perceived as less juicy. Such profiles can be managed through culinary strategies (e.g., brining, marinades, moist-heat cookery) or positioned to consumers who prioritize leanness and high protein. Instrumental metrics captured complementary mechanics (shear vs. TPA), underscoring that tenderness is multi-dimensional. Mechanistically, although WHC was higher and moisture was similar across concepts, the combination of very low intramuscular fat and higher mechanical firmness (higher shear force and first-bite hardness) likely limited lubrication and water release during mastication, resulting in a drier mouthfeel. Colour also contributed to concept separation: darker, redder breast fillets were clearly distinguished by instrumental measures and trained panellists, yet these contrasts did not translate into differences in large-scale consumer *liking appearance*, indicating that visual cues in cooked meat were less influential for consumers than juiciness.

Future work should prioritize factorial designs that control husbandry, pre-slaughter handling and processing conditions while independently testing extensification factors. In addition, applying a cross-over design in which matched subsamples are prepared by both moist- and dry-heat methods can help quantify the impact of cooking on juiciness and tenderness.

## CRediT authorship contribution statement

**Seren Yigitturk:** Writing – original draft, Visualization, Methodology, Investigation, Formal analysis, Data curation, Conceptualization. **Marlene Schou Grønbeck:** Writing – review & editing, Visualization, Methodology, Investigation, Formal analysis, Data curation, Conceptualization. **Shai Barbut:** Writing – review & editing, Supervision, Methodology, Conceptualization. **Line Ahm Mielby:** Writing – review & editing, Investigation, Funding acquisition, Conceptualization. **Birthe Steenberg:** Writing – review & editing. **Sara Wilhelmina Erasmus:** Writing – review & editing, Supervision, Project administration, Methodology, Investigation, Funding acquisition, Conceptualization.

## Disclosures

The authors declare that they have no known competing financial interests or personal relationships that could have appeared to influence the work reported in this paper.

## Appendix Supplementary materials

Image, application 1

Image, application 2
