# Supplementary material for: Can we taste extensiveness? Linking production concepts of extensification factors to the eating quality and consumer liking of chicken breast meat
Source: Poult Sci. 2026 Jan 3;105(3):106379. doi: 10.1016/j.psj.2026.106379 (PMC12819025; doi:10.1016/j.psj.2026.106379)
Supplement: Supplementary file 1 [file mmc1.pdf]

TEKNOLOGISK
INSTITUT

Gregersensvej 9
DK - 2630 Taastrup
Tel. 72 20 20 00

DMRI@teknologisk.dk
www.teknologisk.dk

06.10.2025

To whom it may concern,

Ethics statement

Re. the submitted article "Can we taste extensiveness? Linking production concepts of extensification factors to the eating quality and consumer liking of chicken breast meat"

Danish Technological Instite (DTI) hereby confirms that the submitted article follows ethical principles in research and publishing.

DTI follows ethical rules of scientific research and complies with the guidelines of The Danish Ministry of Higher Education and Science and The Danish Council on Ethics.

The submitted article is based on the mEATquality project (EU Horizon 2020 project #101000344). For this project, DTI carried out a consumer study and a sensory analysis using trained panelists on different pork and chicken products. The ethical concerns for this project are that the participants are provided with the necessary information, and that data is properly managed. This was ensured as follows:

- Panelists were informed in writing about their role and handling of data, and all data was anonymized and reported as a group average. Only the panel leader has access to the original data, which is stored securely for 5 years.
- The participants in the consumer study were given written declarations on confidentiality, anonymity and voluntary participation. Participants could withdraw their participation and their response data at any time. After the survey, the consumers were informed about the purpose of the study.
- All data is handled and stored in full compliance with GDPR rules.

With respect to ethics in publishing, the submitted article follows ethical principles as stated by the lead author.

Sincerely,

Lene Meinert
Director, Ph.D.
Food and Production
Danish Technological Institute
