# Supplementary material for: Can we taste extensiveness? Linking production concepts of extensification factors to the eating quality and consumer liking of chicken breast meat
Source: Poult Sci. 2026 Jan 3;105(3):106379. doi: 10.1016/j.psj.2026.106379 (PMC12819025; doi:10.1016/j.psj.2026.106379)
Supplement: Supplementary file 2 [file mmc2.docx]

**EXTENSIFICATION FACTORS AND EATING QUALITY**

**Can we taste extensiveness? Linking production concepts of extensification factors to the eating quality and consumer liking of chicken breast meat**

Seren Yigitturk^a,1^, Marlene Schou Grønbeck^b,1^, Shai Barbut^c, d^, Line Ahm Mielby^b,^*, Birthe Steenberg^e^, Sara Wilhelmina Erasmus^a,^*

^1^ S. Yigitturk and M.S. Grønbeck share the first authorship of this article.

**Supplementary Material**


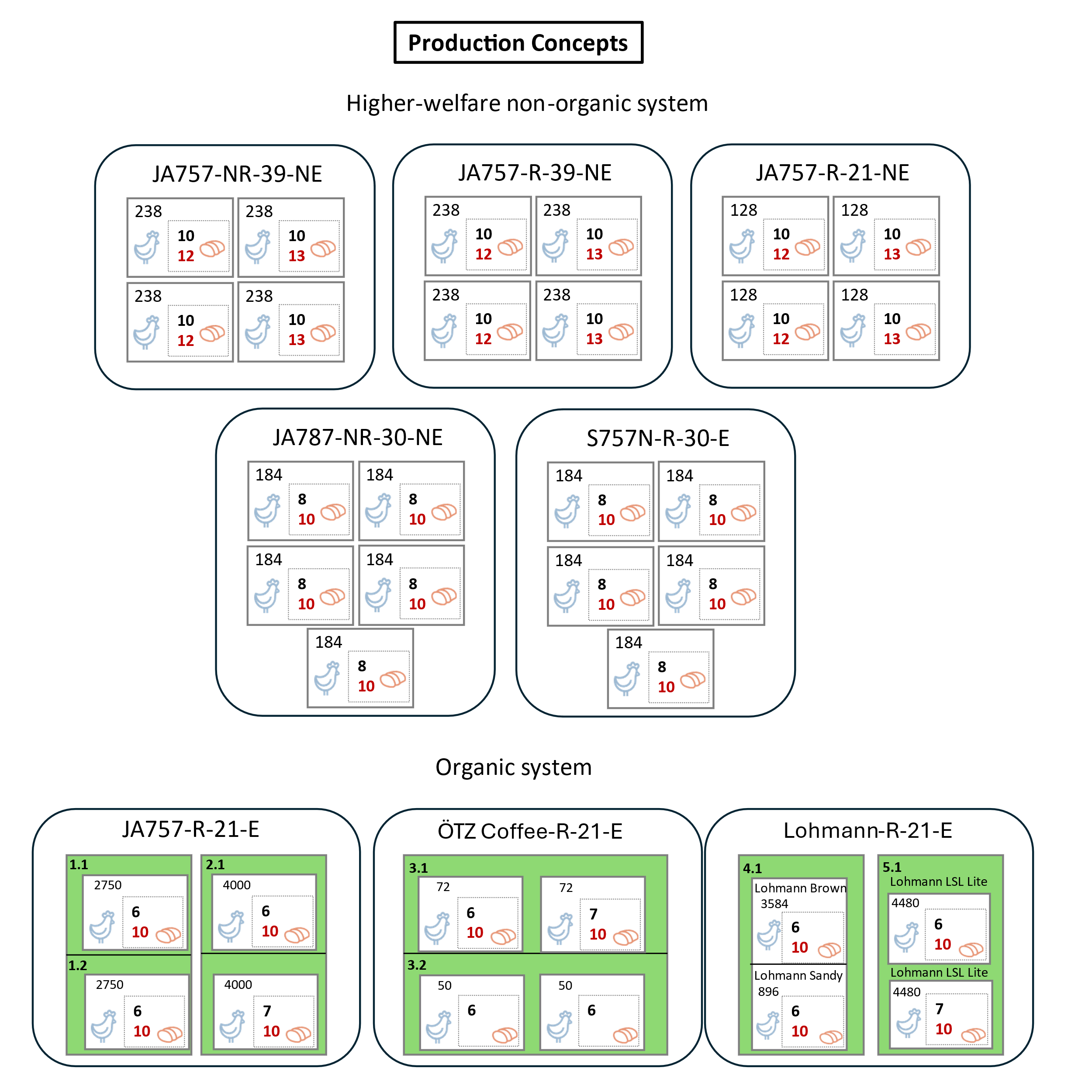


**Supplementary Fig. 1.** **Schematic overview of the experimental structure and sampling design across higher-welfare non-organic and organic production concepts.** The figure illustrates the hierarchical organisation of farms, batches, pens and genetic lines within each concept. For the higher-welfare non-organic system, each production concept comprised a single farm and batch; consequently, pens represented the experimental units for within-concept comparisons. Organic concepts involved multiple farms, batches, pens and, for the Lohmann concept, three genetic lines. Farm and batch identifiers are indicated by the first and second numbers shown, respectively, while pens are visualised within each concept. These hierarchical structures were accounted for by aggregating data at the experimental-unit level, as defined in the *Data Analysis* section. The total number shown in each pen indicates the number of birds housed, while numbers within each pen indicate the number of birds sampled for physicochemical quality analyses (black) and for descriptive sensory analysis and consumer testing (red).


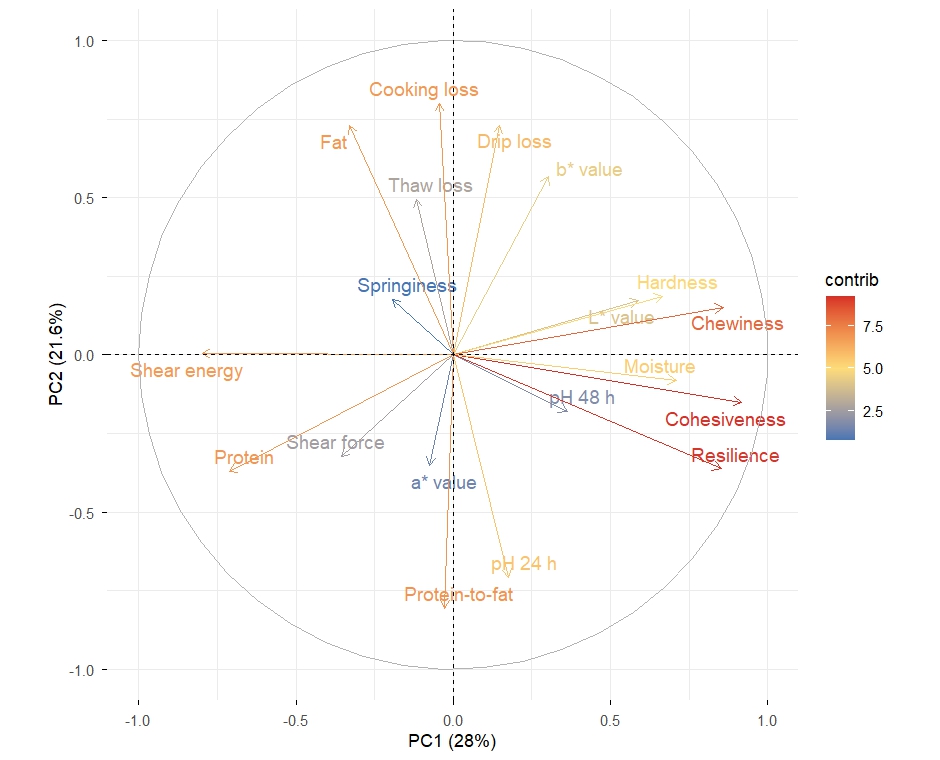


**Supplementary Fig. 2. Principal component analysis (PCA) of physicochemical quality traits**. PCA loading plot of physicochemical quality traits performed at the experimental-unit level. Variables were mean-centred and scaled. Arrow colour indicates each variable’s contribution to PC1 and PC2 (higher contributions shown by warmer colours).
